# The Biological Action and Structural Characterization of Eryngitin 3 and 4, Ribotoxin-like Proteins from *Pleurotus eryngii* Fruiting Bodies

**DOI:** 10.3390/ijms241914435

**Published:** 2023-09-22

**Authors:** Sara Ragucci, Nicola Landi, Lucía Citores, Rosario Iglesias, Rosita Russo, Angela Clemente, Michele Saviano, Paolo Vincenzo Pedone, Angela Chambery, José Miguel Ferreras, Antimo Di Maro

**Affiliations:** 1 Department of Environmental, Biological and Pharmaceutical Sciences and Technologies (DiSTABiF), University of Campania ‘Luigi Vanvitelli’, Via Vivaldi 43, 81100 Caserta, Italy; sara.ragucci@unicampania.it (S.R.); nicola.landi@unicampania.it (N.L.); rosita.russo@unicampania.it (R.R.); angela.clemente@unicampania.it (A.C.); paolovincenzo.pedone@unicampania.it (P.V.P.); angela.chambery@unicampania.it (A.C.); 2 Institute of Crystallography, National Research Council, Via Vivaldi 43, 81100 Caserta, Italy; michele.saviano@cnr.it; 3 Department of Biochemistry and Molecular Biology and Physiology, Faculty of Sciences, University of Valladolid, E-47011 Valladolid, Spain; lucia.citores@uva.es (L.C.); riglesias@uva.es (R.I.); josemiguel.ferreras@uva.es (J.M.F.)

**Keywords:** amino acid sequence, green mold, king trumpet mushroom, MALDI-ToF, ribotoxin-like proteins, sarcin–ricin loop

## Abstract

Ribotoxin-like proteins (RL-Ps) are specific ribonucleases found in mushrooms that are able to cleave a single phosphodiester bond located in the sarcin–ricin loop (SRL) of the large rRNA. The cleaved SRL interacts differently with some ribosomal proteins (P-stalk). This action blocks protein synthesis because the damaged ribosomes are unable to interact with elongation factors. Here, the amino acid sequences of eryngitin 3 and 4, RL-Ps isolated from *Pleurotus eryngii* fruiting bodies, were determined to (i) obtain structural information on this specific ribonuclease family from edible mushrooms and (ii) explore the structural determinants which justify their different biological and antipathogenic activities. Indeed, eryngitin 3 exhibited higher toxicity with respect to eryngitin 4 against tumoral cell lines and model fungi. Structurally, eryngitin 3 and 4 consist of 132 amino acids, most of them identical and exhibiting a single free cysteinyl residue. The amino acidic differences between the two toxins are (i) an additional phenylalanyl residue at the N-terminus of eryngitin 3, not retrieved in eryngitin 4, and (ii) an additional arginyl residue at the C-terminus of eryngitin 4, not retrieved in eryngitin 3. The 3D models of eryngitins show slight differences at the N- and C-terminal regions. In particular, the positive electrostatic surface at the C-terminal of eryngitin 4 is due to the additional arginyl residue not retrieved in eryngitin 3. This additional positive charge could interfere with the binding to the SRL (substrate) or with some ribosomal proteins (P-stalk structure) during substrate recognition.

## 1. Introduction

Ribotoxin-like proteins (RL-Ps) are a family of specific basidiomycetes ribonucleases which catalyze endonucleolytic cleavage of large ribosomal RNA at the level of a specific site located in the sarcin–ricin loop (known as SRL), leading to protein synthesis inhibition and cell death [1]. The specific endonucleolytic action is ascertained by Endo’s assay through the detection of a characteristic fragment of ~450 nucleotides known as the α-fragment when these enzymes are incubated with ribosomes [2]. RL-Ps have been identified and purified from various edible mushroom fruiting bodies such as *Cyclocybe aegerita* (previously known as *Agrocybe aegerita*) [1], *Boletus edulis* [3], *Agaricus bisporus* [4] and two *Pleurotus* species (*P. ostreatus* and *P. eryngii* [5]), most of them are of considerable commercial interest, being a source of high-quality nutrients [6].

Structurally, RL-Ps are monomeric proteins (~15-kDa) with basic pI (usually > 9.0), as displayed by computational analyses [1]. Furthermore, the residues involved in the catalytic action are two aspartyl residues and one histidinyl residue, which constitute the enzymatic catalytic triad [7]. In addition, these enzymes contain a single cysteinyl residue at the N-terminal region, implicated in the binding with metal ions (e.g., Zn^2+^) and protein stability [1].

The cytotoxic effect of RL-Ps likely consists of irreversible damage to the ribosome (in particular, the large subunit), which becomes unable to bind the elongation factors, with consequent arrest of protein synthesis. Indeed, when SRL is damaged, the consequent structural variations also affect the P-stalk, which is usually necessary to allow the correct interaction of elongation factors with ribosomes by enhancing ribosome GTPase activity [8,9]. The structural variations due to the damage at the level of SRL are confirmed by studies on different toxin families targeting SRL, such as ribosome-inactivating proteins from plants (N-glycosylase enzymes) [10,11], Shiga-like toxins from bacteria (analogues to plant N-glycosylases) [12,13] and ribotoxins, specific extracellular ribonucleases isolated from ascomycetes fungi [14,15]. These studies highlight that the variation in the interactions between the P-stalk and these toxins is important to facilitate the recruitment of toxins on the ribosomes, modulating their toxicity [16].

Most RL-Ps exhibit cytotoxic effects towards several human malignant cell lines, while ageritin, the prototype of RL-Ps, isolated from *C. aegerita* fruiting bodies, also exhibits antifungal activity [1] and cytotoxic effect towards insect Sf9 cell line [5]. In this framework, it is reasonable to consider RL-P involvement in fungi self-defence mechanisms, while a possible physiological role must be further investigated.

On the other hand, in applied research, RL-Ps or analogous enzymes such as ribotoxins from ascomycetes could be used in agriculture to control insect pests [17]. Moreover, due to their ability to arrest protein synthesis and trigger apoptotic pathway, RL-Ps could be used in medicine as a toxic part of immunotoxins (i.e., toxins linked to an antibody or its fragment) specifically directed against a target, usually an antigen from a cancer cell promoting specific cell death [18]. Thus, immunotoxins represent an alternative strategy to obtain selective anticancer agents with minimal adverse effects on normal tissues, overcoming the limits of chemotherapy and radiotherapy such as drug resistance development, lack of neoplastic cell selectivity, occurrence of severe side effects and secondary complications [18].

Finally, several bioinformatic approaches performed to find homologous proteins in basidiomycetes showed that the gene of these enzymes is conserved [1]. In particular, the RL-P gene retrieved for ageritin and ostreatin (RL-P isolated from *P. ostreatus*) consists of a coding sequence (CDS) for a pre-protein (pre-form), exhibiting an additional N- and C-terminal amino acid region, not retrieved in native proteins, likely involved in the sorting or controlling their toxicity in the fungi that express these toxins [1].

In this scenario, to obtain more information on the biological and antipathogenic activities as well as structural features of the RL-P family, we decided to further analyse eryngitin 3 and 4 (~15-kDa), novel RL-Ps recently isolated from *P. eryngii* fruiting bodies (known as king trumpet mushroom or ‘Cardoncelli’ in Italian) [5]. Based on the significant differences found in the biological and antipathogenic activities of the two isolated proteins, we carried out an extensive structural characterization to determine the primary structure of both RL-Ps. Finally, the 3D models of both toxins (obtained by AlphaFold prediction) were used to give a possible explanation of the biological differences found between eryngitin 3 and 4.

## 2. Results and Discussion

### 2.1. Purification of Eryngitin 3 and 4

Eryngitin 3 and 4 purification was performed by applying the procedure reported by Landi et al. (2022) [5]. Briefly, a crude extract of *P. eryngii* fruiting bodies was acidified with glacial acetic acid, and the soluble protein fraction was fractionated, taking into account both the differences in protein size (gel filtration chromatography) and the charge characteristics (ion exchange chromatography). In particular, the last chromatographic step (i.e., cation exchange chromatography) allowed the separation of eryngitin 3 and 4 on the basis of charge differences besides their same molecular weight [5].

**Figure 1:**
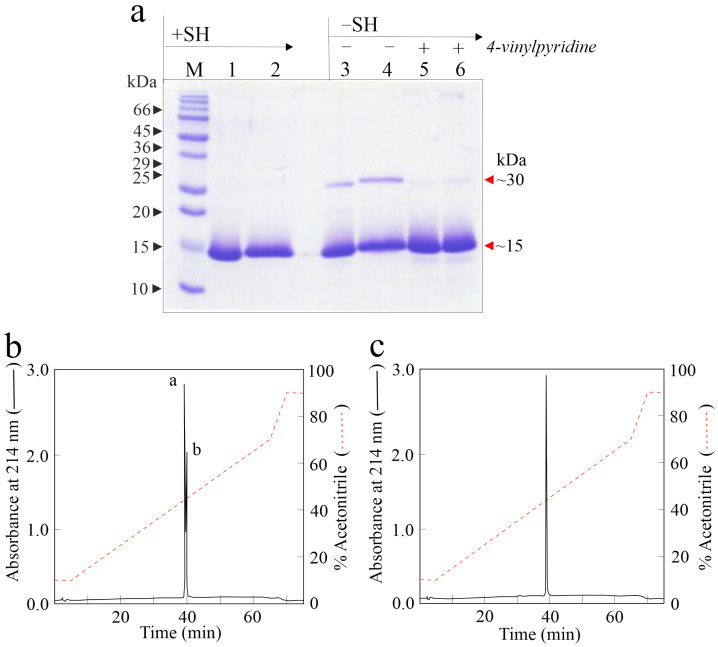
Purification of eryngitin 3 and 4. (**a**) SDS-PAGE analysis of eryngitin 3 and 4 isolated from *P. eryngii* fruiting bodies. SDS-PAGE was carried out in 15% polyacrylamide separating gel. M, molecular markers. A total of 3.0 µg protein was loaded. Eryngitin 3 and 4 were analyzed (i) under reducing conditions (+SH; lanes 1 and 2, respectively), (ii) without reducing agent (−SH; lanes 3 and 4, respectively) and (iii) after alkylation with 4-vinylpyridine without reducing agent (−SH; lanes 5 and 6, respectively). (**b**,**c**) RP-HPLC profiles of native eryngitin 3 and 4, respectively. Typically, 100 µg of protein was injected. For a and b component information in panel B, see Section 3.3 in main text.

The homogeneity of purified proteins was assessed by SDS-PAGE analysis with and without a reducing agent (Figure 1a).

Aliquots of eryngitin 3 and 4 in the presence of a reducing agent (lanes 1 and 2, Figure 1a) showed a single protein band with an electrophoretic migration of ~15-kDa, while without a reducing agent, the presence of a main protein band of ~15-kDa and an additional faint protein band of ~30-kDa (lanes 3 and 4, Figure 1a), for both proteins, is confirmed. Considering the presence of a single free cysteinyl residue responsible for dimeric forms under denaturing conditions in ostreatin, a homologous enzyme previously isolated from *P. ostreatus* fruiting bodies, SDS-PAGE analysis was carried out without reducing agent after treatment with 4-vynylpyridine, a chemical agent able to alkylate free cysteinyl residues. In this condition, the disappearance of the ~30-kDa protein band is evident for both eryngitins (lanes 5 and 6, Figure 1a). In light of this, both eryngitins likely have at least one free cysteinyl reactive residue, as previously reported for ostreatin and ageritin, the latter being a prototype of RL-Ps isolated from *C. aegerita* fruiting bodies [1].

### 2.2. Assessment of Biological and Antipathogenic Activities of Eryngitin 3 and 4

In a previous study, the ability of eryngitin 3 and 4 to release the α-fragment, hallmark of RL-Ps, and their cytotoxic effect towards both human (HUVEC) and insect (Sf9) cell lines were ascertained [5]. These enzymes were able to release the α-fragment and exhibit low in vitro cytotoxicity against the cell lines tested. In this framework, considering their possible involvement in fungal self-defence mechanisms, a further enzymatic characterization (i.e., in vitro IC_50_ determination and α-fragment release) as well as the evaluation of in vitro biological and antipathogenic activities on model cells/organisms were carried out.

**Figure 2:**
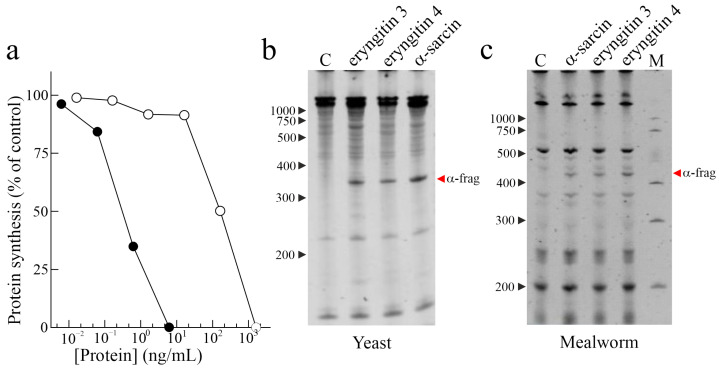
Biological activities of eryngitin 3 and 4. (**a**) Effect of eryngitin 3 (black circles) and eryngitin 4 (white circles) on protein synthesis. Translation assays were carried out using a cell-free system, as indicated in Materials and Methods. (**b**,**c**) Ribonucleolytic action of eryngitin 3 and eryngitin 4 in yeast and mealworm ribosomes, respectively, compared to that of α-sarcin. Each lane contained 5.0 µg (**b**) or 3.0 µg (**c**) of RNA isolated from ribosomes untreated (C, control) or treated with the toxin. The red arrows indicate the RNA α-fragment released as a result of the action of toxins. Numbers indicate the size of the markers (M) in nucleotides.

The determination of the IC_50_ (concentration inhibiting 50% of protein synthesis) of eryngitin 3 and 4 by in vitro assay was evaluated using a rabbit reticulocyte lysate system. Considering that the α-fragment (Endo’s assay) release is only a qualitative result, IC_50_ determination is important to obtain the quantitative information necessary to compare the different toxins. In particular, eryngitin 3 and 4 exhibit an IC_50_ of 0.3 ng/mL (20.7 pM) and 160 ng/mL (11.0 nM), respectively (Figure 2a). Thus, under the same experimental conditions, the IC_50_ value of eryngitin 3 is ~530-fold lower compared to that of eryngitin 4, although both toxins release the α-fragment. Moreover, the IC_50_ value of eryngitin 3 is 6.5-fold lower than that of ageritin (IC_50_ = 133 pM), a prototype of RL-Ps isolated from *C. aegerita* fruiting bodies and 11.3-fold lower than that of ostreatin (IC_50_ = 234 pM), a homologous RL-P isolated from *P. ostreatus* fruiting bodies [1]. Subsequently, to investigate whether eryngitin 3 and 4 had specific ribonuclease action on the SRL, we carried out the Endo’s assay on yeast (*Saccharomyces cerevisiae* L.) and mealworm insect (*Tenebrio molitor* L.) ribosomes. As shown in Figure 2b,c, when ribosomes were incubated with eryngitin 3 and 4, the characteristic α-fragment, identical to that produced by the ribotoxin α-sarcin [1], was produced. This clearly indicates that eryngitin 3 and 4 exhibit a specific ribonuclease action on ribosomes from different sources. On the other hand, this suggests that these proteins could play an insecticidal and fungicidal role, as has been suggested for ribotoxins [5,14,15].

**Figure 3:**
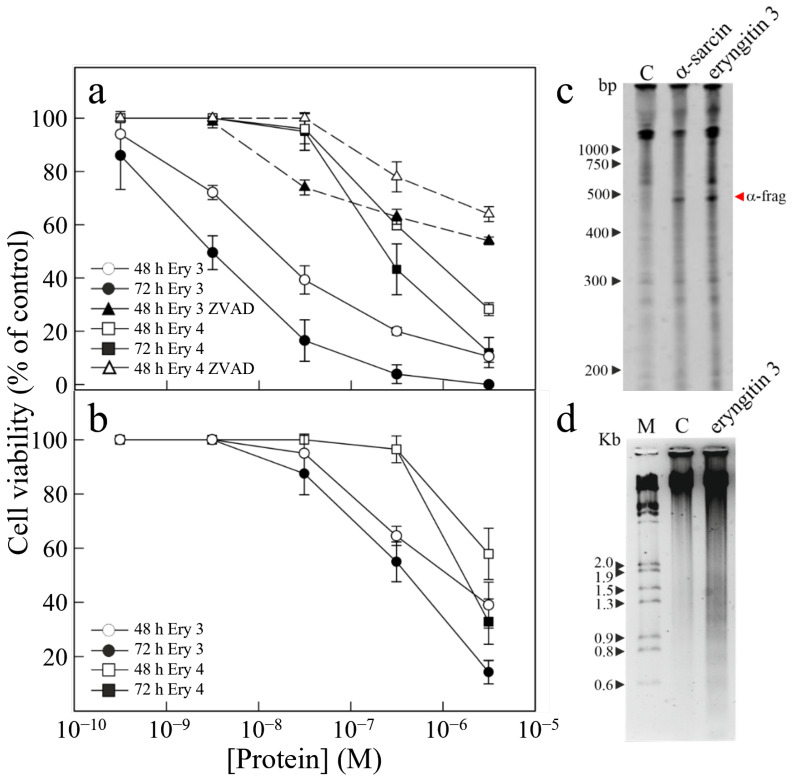
Cytotoxic effect of eryngitins 3 and 4 against human malignant cells. (**a**,**b**) Effect of eryngitin 3 (Ery 3) or eryngitin 4 (Ery 4) on the viability of HeLa and COLO 320 cells, respectively. Cells were grown in RPMI 1640 medium and incubated with different eryngitin concentrations for 48 h and 72 h, and cell viability was evaluated by a colorimetric assay. To investigate the effect of Z-VAD on the viability of HeLa cells, the cells were preincubated for 3 h with Z-VAD and then incubated with different concentrations of eryngitin 3 or 4 for 48 h, and cell viability was evaluated. Data represent the mean ± SD of two experiments performed in duplicate. (**c**) Ribonucleolytic action of eryngitin 3 on RNA from HeLa cells. Each lane contained 1.0 μg of RNA isolated from either untreated cells (C, control) or cells incubated with 30 nM of α-sarcin or eryngitin 3 for 72 h. Red arrow indicates the α-fragment released as a result of specific ribonuclease action. Numbers indicate the size of the standards (M) in nucleotides. (**d**) Effect of eryngitin 3 on internucleosomal DNA fragmentation. COLO 320 cells were incubated in the absence (C) or presence of 0.3 µM of eryngitin 3 for 72 h. The DNA was isolated, and 4.0 µg was electrophoresed, as indicated in Materials and Methods. The numbers indicate the corresponding size of the standards (M) (λDNA HindIII/EcoRI) in Kb.

In addition, to obtain more information on their cytotoxic effects against different human tumour cell lines, eryngitin 3 and 4 were tested on both HeLa and COLO 320 cell lines, and the IC_50_ values (concentration of protein causing 50% of cell death) of eryngitin 3 and 4 are reported in Table S1. As displayed in Figure 3a, the most sensitive were HeLa cells, with IC_50_ values from 3.0 to 230 nM after 72 h of treatment, while COLO 320 cells have values between 420 to 1700 nM after 72 h of treatment (Figure 3b). The lowest IC_50_ values for both cell lines refer to eryngitin 3, which is 77-fold and 4-fold more toxic than eryngitin 4 when incubated with Hela and COLO 320 cells, respectively. In previous works, the RL-Ps cytotoxicity was attributed to their ability to trigger the apoptotic pathway [1] that may be linked to signalling through the ribotoxic stress response or to inhibition of protein synthesis. Therefore, we first wanted to see whether, after endocytosis, eryngitin 3 was able to reach the cytosol and inactivate the ribosomes. Thus, we analyzed the rRNA from HeLa cells treated with eryngitin 3 for 48 h. Figure 3c shows that the RL-P displayed 28S rRNA ribonuclease activity on cell ribosomes, as indicated by the release of the α-fragment, demonstrating that eryngitin 3 was able to reach and damage the ribosomes. Second, in order to ascertain the involvement of caspase-mediated apoptosis in eryngitin-3- and 4-mediated cell death, we tested the sensitivity to the pan-caspase inhibitor Z-VAD for HeLa cells or the cleavage of chromosomal DNA into oligonucleosomal fragments for COLO 320 cells.

In particular, HeLa cells were pre-treated and maintained in 100 µM Z-VAD for 48 h, and cell viability was determined in the presence of different concentrations of eryngitin 3 and 4. As shown in Figure 3a, the presence of Z-VAD increased cell viability from 11 to 54% in 3.1 μM eryngitin-3-treated cells and from 29 to 64% in 3.1 μM eryngitin-4-treated cells. Moreover, the typical apoptotic morphological features were also visualized microscopically when HeLa cells were incubated with eryngitin 3 or eryngitin 4 for 48 h (Figure S1). On the other hand, when COLO 320 cells were treated for 72 h with 300 nM of eryngitin 3, the breakdown of nuclear DNA into oligonucleosomal fragments was clearly observed (Figure 3d). Altogether, our data suggested that the apoptotic pathway was involved in cell death mediated by the ribonucleolytic activity of eryngitin 3 and 4, as already proved for other RL-Ps [1].

**Figure 4:**
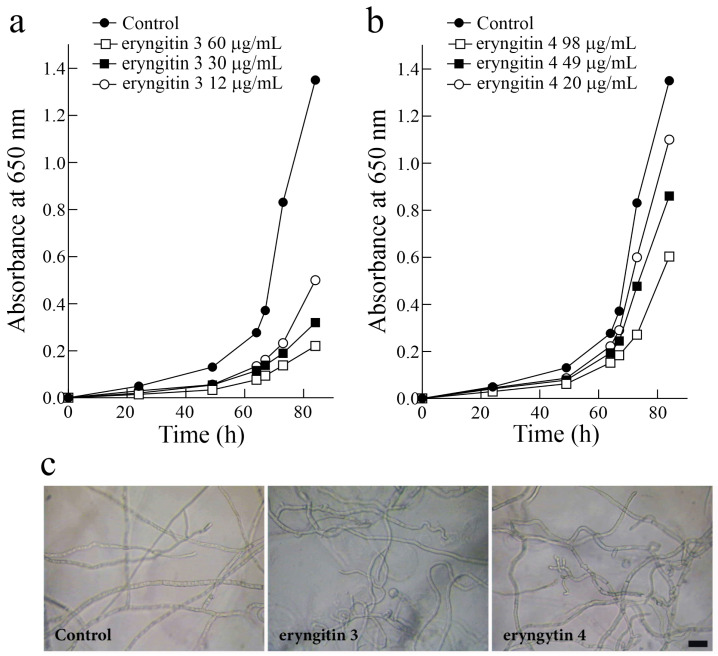
Antifungal activity. The effect of eryngitin 3 (**a**) and 4 (**b**) against *Penicillium digitatum* was measured in a microtiter plate bioassay. Conidia of *P. digitatum* were grown in potato dextrose broth (PDB) at 28 °C in the presence of different eryngitin concentrations. Fungal growth was followed for 84 h and measured as an increase in absorbance at 650 nm. The curves represent the buffer control or different amounts (µg/mL) of both toxins. One representative experiment performed in triplicate is shown. (**c**) Morphological changes in *P. digitatum* mycelium exposed to eryngitins. The *P. digitatum* mycelium was grown in the absence (control) or presence of 60 μg/mL eryngitin 3 or 98 μg/mL eryngitin 4. After incubation for 48 h, samples were visualized using light microscopy at 200× magnification. Bar, 5 µm.

Finally, we evaluated the effects of eryngitin 3 and 4 on the growth of the green mold *Penicillium digitatum*. This ascomycete fungus is responsible for the postharvest decay of citrus, causing a significant reduction in citrus quality and marketable yield [19]. As shown in Figure 4, eryngitin 3 and 4 exhibit a growth-inhibitory effect on *P. digitatum* in a concentration-dependent manner.

In particular, 60, 30 and 12 µg/mL of eryngitin 3 resulted in 84%, 76% and 66% growth inhibition, respectively, after 84 h of growth (Figure 4a). On the contrary, eryngitin 4 showed less ability to inhibit *P. digitatum* growth. Indeed, fungal growth was inhibited by 53%, 36% and 18% after 84 h using 98, 49 and 20 µg/mL of eryngitin 4, respectively, which are ~1.6-fold higher concentrations with respect to those of eryngitin 3 (Figure 4b). Furthermore, the toxicity of eryngitins on the mycelial growth of *P. digitatum* was also visualized microscopically by observing alterations in hyphal morphology (Figure 4c).

Taken together, both biological and antipathogenic data evidence a more pronounced toxic effect of eryngitin 3 compared to eryngitin 4, although both enzymes are able to release the α-fragment, making necessary a complete structural characterization to justify their functional differences.

### 2.3. Relative Molecular Masses of Eryngitin 3 and 4 with and without Alkylation

In order to achieve the relative molecular masses (M*r*) of native eryngitin 3 and 4, as well as their cysteinyl residue/s content, M*r* of both native and alkylated proteins after RP-HPLC desalting was determined by MALDI-ToF MS analyses.

**Figure 5:**
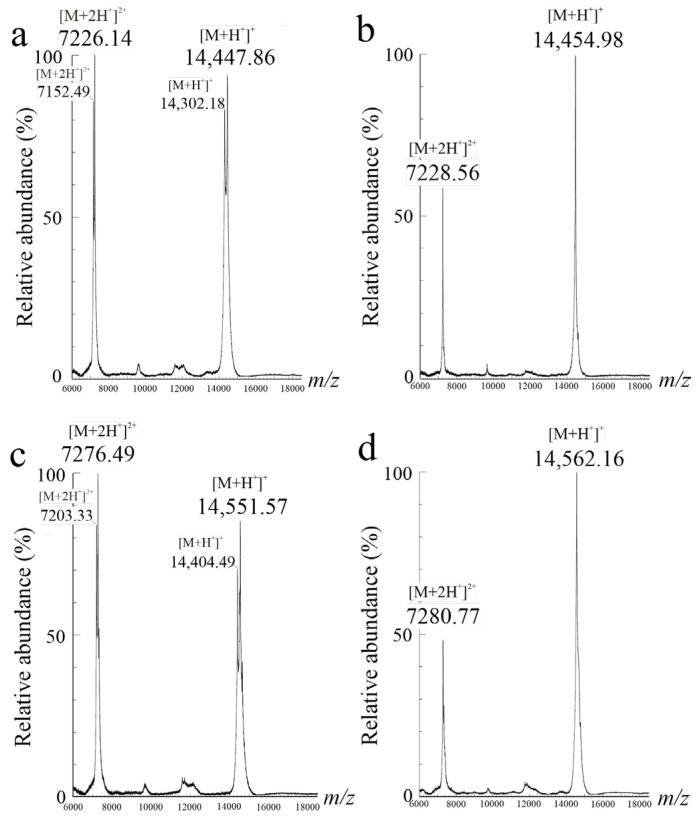
MALDI-ToF mass spectra of native and alkylated eryngitin 3 and 4. (**a**,**b**), MALDI-ToF spectra of native eryngitin 3 and 4, respectively. (**c**) and (**d**), MALDI-ToF spectra of alkylated eryngitin 3 and 4, respectively. [M+H^+^]^+^ and [M+2H^+^]^2+^ correspond to the single and doubly charged ions, respectively.

The chromatographic profiles of native eryngitin 3 and 4 are reported in Figure 1b,c, respectively. RP-HPLC highlights a heterogeneity for eryngitin 3 preparation, not detected by SDS-PAGE analysis, characterized by the elution of two overlapping protein peaks (Figure 1b; component a~68%, component b~32%) and a single protein peak for eryngitin 4 (Figure 1c), confirming the homogeneity found by SDS-PAGE analysis for the latter. Moreover, considering the difficulty in separating the two components of eryngitin 3 by RP-HPLC after several attempts of elution conditions, we decided to directly analyze both components a and b of eryngitin 3 in a mixture after RP-HPLC, as well as the single peak of eryngitin 4 by MALDI-ToF MS. Typical MALDI-ToF MS spectra of eryngitin 3 mixture and eryngitin 4 are shown in Figure 5a,b, respectively.

The mixture of two overlapping eryngitin 3 peaks (components a and b) contained two experimental M*r*, 14,447.86 ([M+H^+^]^+^) and 14,302.18 ([M+H^+^]^+^), while a single M*r* of 14,454.98 ([M+H^+^]^+^) was retrieved for eryngitin 4. In particular, a difference of ~146-Da (14,447.86 – 14,302.18 = ~146-Da) between components a and b was found in the eryngitin 3 mixture. Subsequently, the experimental M*r* of eryngitin 3 and 4 were acquired after reduction and alkylation with 4-vinylpyridine, and the related MALDI-ToF MS spectra are displayed in Figure 5c,d. In these conditions, the experimental M*r* of both components detected in the eryngitin 3 mixture (components a and b after RP-HPLC) and the M*r* of eryngitin 4 differed by ~105-Da compared to the experimental M*r* of native proteins. This additional molecular mass of ~105-Da corresponds to the chemical modification of a single cysteinyl residue to pyridyl-cysteine in the presence of 4-vinylpyridine [20]. In addition, similar results were also obtained when the proteins were directly subjected to alkylation with 4-vinylpyridine without preliminary reduction. Indeed, both eryngitin 3 components and eryngitin 4 exhibit an additional molecular mass of ~105-Da.

Overall, these results suggest that both components of eryngitin 3, as well as eryngitin 4, contain a single free cysteinyl residue susceptible to *S*-pyridylethylation (Δ-mass = 105-Da). This structural characterization suggests that the covalent dimeric forms retrieved in SDS-PAGE without a reducing agent are due to the formation of interchain disulphide bonds under denaturing conditions.

### 2.4. N-Terminal Amino Acid Determination and Search in Fungal Data Base Genome

In order to clarify the structural features which could explain the differences in biological and antipathogenic activities of eryngitin 3 and 4, we decided to get the amino acid sequence of both proteins. Therefore, to optimize the information reported in the fungal genomics resource ‘MycoCosm’ online database [21], the following strategy was carried out: (i) N-terminal amino acid sequence determination by using Edman degradation; (ii) search of similar proteins in *P. eryngii* genome by using N-terminal sequence as query; (iii) enzymatic cleavage with endoproteinases (i.e., trypsin, endoproteinase Glu-C, chymotrypsin, and pepsin) followed by MALDI-ToF MS analysis of the resulting peptides; and (iv) mapping of the obtained peptides with a homologous reference protein.

**Figure 6:**
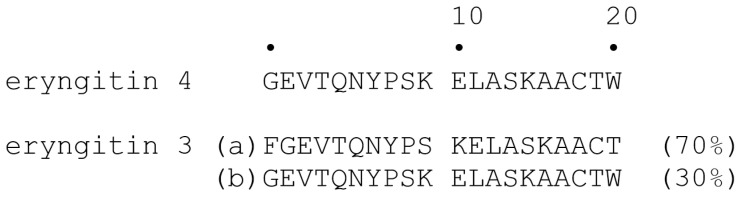
N-terminal amino acid sequences of alkylated eryngitin 4 and eryngitin 3 obtained by automatic Edman degradation. Eryngitin 3 exhibit two amino acid sequences (a and b) assigned on the basis of their different amount.

Automatic Edman degradation of eryngitin 4 and 3 mixture after RP-HPLC provided a single N-terminal sequence for eryngitin 4 and two different amino acid sequences for eryngitin 3 (the two N-terminal amino acid sequences were assigned on the basis of their different amount) [22]. N-terminal amino acid sequences up to 20 residues are reported in Figure 6.

These data show that most of the amino acid residues constituting eryngitin 4 N-terminal sequence and the two N-terminal sequences of eryngitin 3 are identical. In particular, the N-terminal of eryngitin 4 and the N-terminal amino acid sequence that are present in a lower amount (30%; component b) in the eryngitin 3 mixture are identical, while the N-terminal sequence present in a higher amount (70%; component a) in the eryngitin 3 mixture has an additional phenylalanyl residue at the N-terminal region.

Subsequently, the N-terminal amino acid sequence of eryngitin 3 (1-FGEVTQNYPS KELASKAACT-20) was used as a query for protein sequence identification in the *P. eryngii* genome by using the BlastP tool. The algorithm identified a hypothetical protein BDN71DRAFT_1455417 (AC: KAF9489889.1; 162 amino acid residues) (Figure S2), which showed the same N-terminal amino acid sequence of both eryngitin 3 and 4. In this framework, we decided to use the amino acid sequence of this hypothetical protein as a reference protein for the determination of eryngitin 4 and 3 amino acid sequences.

### 2.5. Determination of Eryngitin 4 Amino Acid Sequence

Considering the high homogeneity of eryngitin 4, we first determined its amino acid sequence by MALDI-ToF MS analysis followed by peptide mapping strategy using the hypothetical protein BDN71DRAFT_1455417 as reference protein (162 amino acid residues; Figure S2), which exhibits 100% identity with the first 20 amino acid residues of alkylated eryngitin 4.

**Figure 7:**
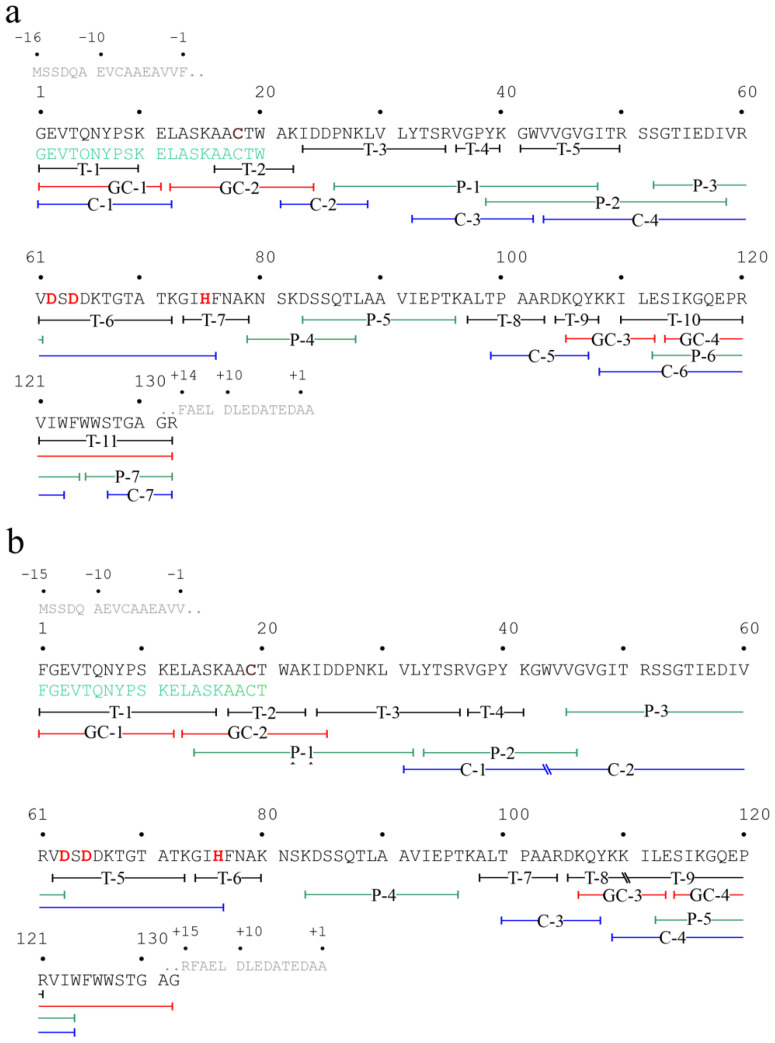
Amino acid sequence of eryngitin 4 and 3. The overlapping peptides obtained by digestions of alkylated eryngitin 4 (**a**) and eryngitin 3 (**b**) used to confirm the amino acid sequence of uncharacterized protein from *Pleurotus eryngii* ATCC 90797 (AC: KAF9489889.1) are reported. Grey represents N- and C-terminal additional amino acid residues found in reference protein retrieved in *P. eryngii* genome (Figure S2). The standard one-letter code was used for the amino acid residues. Catalytic residues are highlighted in red; free cysteinyl residues are in violet. Abbreviations: C, GC, P and T represent chymotrypsin, endoproteinase Glu-C, pepsin and tryptic peptides, respectively.

In light of this, a first trypsin mapping on alkylated eryngitin 4 was carried out. Tryptic peptide molecular masses retrieved in digested samples are reported in Table S2, whereas their sequence positions are mapped in Figure 7a. This set of data provided for 67.8% of reference proteins (99 out of 146 amino acids, considering Gly17 and Ala162 as the first and last amino acid residues at N- and C-terminus of hypothetical protein BDN71DRAFT_1455417, respectively (Figure S2). Notably, no tryptic peptide mapping of the reference protein in the C-terminal region was found. Thus, for sequence completion and/or verification, we decided to perform a complete peptide mapping by MALDI-ToF mass spectrometry, with new peptide sets of alkylated eryngitin 4 derived from endoproteinase Glu-C, chymotrypsin and pepsin digestion. These new peptide sets are reported in Table S2 and mapped in Figure 7a. Overall, all obtained peptides overlap the entire amino acid sequence of the reference protein from Gly17 to Arg148. Thus, the results exclude that native eryngitin 4 has the amino acid regions at the N- (position 1–16) and C-terminal (position 149–162) regions, like the reference protein, confirming that eryngitin 4 consists of 132 amino acids (Figure 7a).

Finally, the agreement between the experimental mass of the native protein (Figure 5b; 14,454.98 Da; [M+H^+^]^+^) and the calculated one, on the basis of the amino acid sequence (14,455.38 Da), confirms the correctness of the amino acid sequence determination. Moreover, eryngitin 4 preserves the catalytic triad of RL-Ps: Asp62, Asp64 and His75 assigned by homology, considering the experimental data reported for ageritin, a prototype of RL-Ps family [7].

### 2.6. Determination of Eryngitin 3 Amino Acid Sequence

The same strategy reported for the determination of eryngitin 4 amino acid sequence was also carried out to acquire the primary structure of eryngitin 3, considering that the principal component of eryngitin 3 (component a, ~70%) has as first/additional amino acid, a phenylalanyl residue at the N-terminus, as retrieved by Edman degradation, also present at the N-terminal region of reference protein (hypothetical protein BDN71DRAFT_1455417), as shown in Figure S2.

Tryptic mapping of alkylated eryngitin 3 (M*r* peptides set reported in Table S3, and sequence positions are mapped in Figure 7b) provided for 58.2% of the reference protein [85 out of 146 amino acids, considering Phe16 and Ala162 as the first and last amino acid residue at the N- and C-terminus of the hypothetical protein BDN71DRAFT_1455417, respectively (Figure S2)]. Notably, this first data set highlights (i) the absence of tryptic peptides to map the C-terminal region of the reference protein and (ii) the presence of a T1 peptide with a M*r* of 1798.25 ([M+H^+^]^+^) that overlaps with the sequence at position 1–16, confirming the additional phenylalanyl residue at the N-terminus of eryngitin 3 (Figure 7b). Moreover, a peptide with an experimental M*r* of 1122.59 (Table S3), an alternative N-terminus starting with a glycinyl residue (position 2–11 in eryngitin 3, N-terminal peptide of component b), was also detected, confirming that eryngitin 3 is a mixture of two proteins which differ in amino acid at the N-terminus. Indeed, the molecular weight of a phenylalanyl residue is 147.18 Da, corresponding to the molecular weight difference found between components a and b of eryngitin 3 detected after separation by RP-HPLC and MALDI ToF MS analysis (Figure 5c; 14,447.86 − 14,302.18 = ~146 Δ-mass).

However, for eryngitin 3 sequence completion and/or verification, a complete peptide mapping by MALDI-ToF mass spectrometry, with new peptides set of alkylated eryngitin 3 derived from endoproteinase Glu-C, chymotrypsin and pepsin digestion, was performed. The novel different sets of peptides obtained are reported in Table S3 and mapped in Figure 7b. Overall, all peptides obtained overlap with the entire amino acid sequence of the reference protein, from Phe16 to Gly147 (Figure S2). Thus, the results achieved show that eryngitin 3 lacks the additional amino acid regions at the N- (position 1–15) and C-terminus (position 148–162) of the reference protein (Figure S2), while the component retrieved after RP-HPLC analysis differs only by the presence (component a, ~70%) or absence (component b, ~30%) of a phenylalanyl residue at the N-terminus.

Finally, the agreement between experimental M*r* of the native protein (component a, 14,447.86 Da; component b, 14,302.18 Da) and that calculated from the amino acid sequence (component a, 14,446.36 Da; component b, 14,299.19) confirms the correctness of the analysis.

In light of this, it is reasonable to assume that the two eryngitins could be different proteolytic products derived from the same mRNA, considering the presence of a single CDS in the *P. eryngii* genome (see Section 2.7), in order to regulate their activity or function [23].

### 2.7. Features of Eryngitin 3 and 4 Gene

The amino acid sequence of the hypothetical protein BDN71DRAFT_1455417 (AC: KAF9489889.1) was obtained by analyzing the *P. eryngii* ATCC 90797 genome [24]. In particular, the genomic sequence (831 bp) is reported in the MycoCosm database as the “Pleery1_1455417” ID gene. The transcript sequence (730 bp) contains the coding sequence (CDS; 489 bp) of the hypothetical protein KAF9489889.1, showing additional N- and C-terminal amino acid sequences with respect to both native eryngitins and two introns (position 165–216 and 249–297) at 5′ transcript region (Figure S3).

The translation of this transcript covers the entire structure of both purified eryngitins (132 amino acids), including two additional amino acid sequences of ~15 and ~14 at the N- and C-terminus, respectively, compared to purified proteins (Figure S3). This result is consistent with previous data obtained for both ostreatin and ageritin [1], RL-Ps isolated from *P. ostreatus* and *C. aegerita* fruiting bodies, respectively, confirming that these toxins are likely synthesized as pre-forms, which includes both amino acid regions (signal peptides), involved in toxin regulation or subcellular localization, and subsequently removed by specific proteases [25].

Finally, the transcript sequence of eryngitins exhibits two short introns (intron 1, 52 bp; intron 2, 49 bp) located at 5′ CDS region with canonical splice junctions (Figure S3), as reported for most of the mushrooms’ genes [26].

### 2.8. Sequence Comparison between Eryngitin 3 and 4 and Other Well-Characterized RL-Ps

The alignment between eryngitin 4 and eryngitin 3, as well as ostreatin (RL-P from *P. ostreatus* fruiting bodies) and ageritin (RL-P from *C. aegerita* fruiting bodies), was displayed in Figure S4. They are well-characterized RL-Ps, and all amino acid sequences conserve the following invariant amino acid residues: (i) three amino acids forming the catalytic site (Asp69, Asp71 and His82; numbering refers to the alignment consensus sequence); (ii) one cysteinyl residue (Cys19) at the N-terminal region; and (iii) three tryptophanyl residues (W130, W132 and W133) at the C-terminal region. The latter is a structural feature of this protein family, as already highlighted by previous homology studies using ageritin as a query in protein database searching [1]. Furthermore, the identity between eryngitins and ostreatin, isolated from the same genus (*Pleurotus*), is 97.7% (~98.5% similarity), while the identity between eryngitins and ageritin (*Cyclocybe* genus) is 40.1% (~45.4% similarity). This observation highlights the structural differences among RL-Ps found in edible mushrooms belonging to different basidiomycetes genera, which exhibit peculiar enzymatic action/toxicity towards several cell lines or fungal model systems, such as *Penicillium digitatum* [1].

### 2.9. Modeling the 3D Structure of Eryngitin 4 and 3

**Figure 8:**
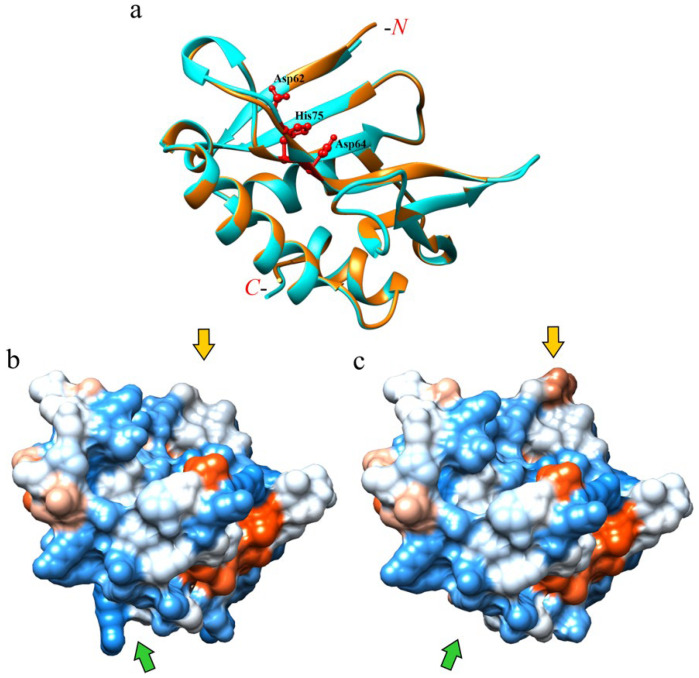
Structural features of eryngitin 3 and 4 by 3D molecular model. (**a**) Superimposed 3D molecular model of eryngitin 4 (cyan ribbon) and eryngitin 3 (orange ribbon) obtained by AlphaFold software. Stick representation highlights the catalytic triad (Asp62, Asp64 and His75; numbering refers to the eryngitin 4 amino acid sequence). (**b**,**c**) Electrostatics surfaces of eryngitin 4 and 3, respectively. Blue and red show positive and negative surface regions, respectively. Yellow and green arrows highlight the N- and C-terminal regions of the two proteins, respectively. The disposition of 3D models in (**b**,**c**) is in the same molecular orientation as the superimposed models shown in (**a**).

In order to gain insight into the structural differences in the biological and antipathogenic activities of both eryngitins, a structure prediction study was carried out by using AlphaFold Structure Prediction Database (https://www.alphafold.ebi.ac.uk/, accessed on 9 May 2023). As expected, the superimposed 3D models of eryngitin 4 and 3 exhibit the same protein fold (Figure 8a), considering that the two proteins are constituted by the same amino acid sequence, apart from a single residue at both the C-terminus (additional arginyl residue for eryngitin 4 (Arg132), Figure 7a) and the N-terminus (additional phenylalanyl residue for eryngitin 3 (Phe1), Figure 7b).

Moreover, the graphic of the ‘Local Distance Difference Test’ (lDDT), a superposition-free score used to evaluate the local distance differences of all atoms in a model [27], displays a value higher than 90 for both eryngitins models, except for positions 62–78 and the first N- and C-terminus residues (Figure S5). These 3D models consist of an extended antiparallel β-sheet with an interface in which the catalytic triad (Asp62, Asp64 and His75; numbering refers to eryngitin 4) is conserved and an opposite interface resting on four principal α-helixes (α-helix Lys10—Ala21; α-helix Glu93—Ala97; α-helix P100—Ile114; and α-helix P119—Thr128; numbering refers to eryngitin 4), which form the bulk of the main protein fold. In particular, it is interesting to note that the main region with evidently low IDDT (50 < IDDT < 70, positions 62–78) corresponds to the catalytic site of these enzymes, likely confirming that the catalytic site of enzymes is usually characterized by intrinsic conformational freedom that is necessary to adapt to the substrate [28]. Slight structural differences between eryngitin 4 and 3 are evident only at the N-terminal and C-terminal regions, as shown by electrostatic surface representation for the two different 3D models (Figure 8b for eryngitin 4 and Figure 8c for eryngitin 3). Indeed, an accentuated positive electrostatic surface was found at the C-terminal region for eryngitin 4 due to the presence of an additional arginyl residue, while a slightly negative electrostatic surface was found at the N-terminal region of eryngitin 3 due to the presence of an additional phenylalanyl residue.

This observation could explain the functional differences retrieved between the two proteins. Indeed, as highlighted by several structural/functional studies, analogous toxins, such as (i) Shiga-like toxins (bacterial toxins) [29,30], (ii) ribotoxins (ascomycetes toxins) [31], and (iii) ribosome-inactivating proteins (plant toxins) [29,32], sharing the same target (SRL) of eryngitins, exhibit different functional action due to their interaction and/or binding capacity with both SRL and nearby macromolecules [33]. In particular, the macromolecules which can interfere with or enhance the binding between these toxins and SRL are ribosomal proteins, generally forming the ribosomal stalk [16,30], a specific ribosomal structure required for the binding of elongation factors to the ribosomes during protein translation [34,35]. In this context, it can be hypothesized that slight electrostatic surface changes at the N- and C-terminal regions could justify the different functional features of the two eryngitins by altering their interaction with SRL, the site where they exploit their specific ribonuclease activity.

In addition, some experimental data led us to consider that the involvement of slight structural variations at the N- and C-terminal regions may account for the differences in biological and antipathogenic activities of both eryngitins. Indeed, although the two enzymes share the same structural fold, eryngitin 3 exhibits a higher thermal stability (Tm = ~89.7 °C) compared to eryngitin 4 (Tm = ~83.1 °C) [5]. Furthermore, characterization of the ageritin isoform, named Met-ageritin (homologous to eryngitins), with an additional N-terminal methionyl residue compared to ageritin, showed that this single additional amino acid residue at the N-terminus drastically decreased Met-ageritin enzymatic action [1]. Finally, the single additional positive charge of eryngitin 4 due to the arginyl residue at the C-terminus affects its physicochemical characteristics, considering a longer retention time of eryngitin 4 compared to eryngitin 3 during the last purification step (ion exchange chromatography, which separates proteins on the basis of charge differences) [5].

## 3. Materials and Methods

### 3.1. Chemicals and Reagents

The chemicals used in this work were previously reported [5,22], and most of them were obtained from Sigma-Aldrich Solutions (Merk Life Science, Milan, Italy). Endoproteinases (trypsin TPCK-treated, chymotrypsin, endoproteinase Glu-C and pepsin) for protein digestion were purchased from Sigma-Aldrich Solutions (Merk Life Science, Milan, Italy). Century™-Plus RNA Markers were purchased from Fisher Scientific (Madrid, Spain). Potato dextrose agar and potato dextrose broth media were purchased from Sigma-Aldrich (Madrid, Spain). The RPMI 1640 medium, fetal bovine serum (FBS), penicillin, streptomycin and trypsin were purchased from GIBCO BRL (Barcelona, Spain). The Z-VAD-fmk (pan-caspase inhibitor carbobenzoxy-valyl-alanyl-aspartyl-[O-methyl]-fluoromethylketone) named ZVAD was purchased from R&D Systems (Abingdon, UK). GelRed was purchased from Biotium Inc. (Hayward, CA, USA).

The mealworms (*Tenebrio molitor* L.) were bought at local markets. The strain of *Penicillium digitatum* (Pers.) Sacc. was obtained by the Spanish Type Culture Collection (CECT), Valencia, Spain. COLO 320 (human colon adenocarcinoma) and HeLa (human cervix epitheloid carcinoma) cell lines used in this study were obtained from the European Collection of Cell Cultures (ECACC) and grown in RPMI 1640 medium (GIBCO BRL, Barcelona, Spain) supplemented with 10% fetal bovine serum (FBS), 100 U/mL penicillin and 0.1 mg/mL streptomycin under 5% CO_2_ at 37 °C.

### 3.2. Proteins Purification

The purification of eryngitin 3 and 4 for biological assessment and structural characterization was achieved, as previously reported [5]. Briefly, *P. eryngii* fruiting bodies extract was subjected to acetic acid precipitation at pH 4.0, and soluble proteins were separated, exploiting the differences in both protein size (size exclusion chromatography) and charge (cation exchange chromatography) [1].

### 3.3. Analytical Procedures

Purity and integrity of eryngitin 3 and 4 were determined by SDS-PAGE [36] with a Mini-Protean II mini-gel apparatus (Bio-Rad, Milan, Italy) using 6% stacking and 15% separation polyacrylamide gel; a precision plus protein kit (Bio-Rad, Hercules, CA, USA) was used for reference proteins. Protein concentration was determined by Pierce BCA Protein Assay kit (Thermo Fisher Scientific, Rodano, Italy), using BSA as standard [37].

### 3.4. Assays of Cell-Free Protein Synthesis

The effect of eryngitin 3 and 4 on protein synthesis was determined through a coupled transcription–translation in vitro assay using a rabbit reticulocyte lysate system (Promega, Alcobendas, Madrid, Spain), as previously reported [38]. Data represent the mean of three experiments in triplicate.

### 3.5. Ribonucleolytic Activity on Yeast and Mealworm Ribosomes

The specific activity of ribotoxins, manifested by the release of α-fragment from rRNA after enzymatic action on yeast and mealworm ribosomes, was detected as described elsewhere [5,38]. The 30,000× *g* supernatants (partially purified ribosomes, named S30) from yeast and mealworm (used as substrates) were obtained as described elsewhere [38,39].

### 3.6. Cell Viability Assays

Cell viability was determined with a colorimetric assay based on the cleavage of tetrazolium salt WST-1 to formazan by mitochondrial dehydrogenases in viable cells, as described elsewhere [38]. The concentration of toxins causing a 50% reduction in viability (IC_50_) was calculated by linear regression analysis. Eryngitin 3 and 4 toxicity was also evaluated using HeLa cells pre-treated with 100 µM of the pan-caspase inhibitor Z-VAD. The reagent was added to cells 3 h before toxin administration, and cell viability was determined in the presence of different toxin concentrations.

### 3.7. RNA Extraction from HeLa Cells

HeLa cells (1 × 10^6^/plate) were incubated for 72 h in presence of 30 nM eryngitin 3. After treatment, cells were harvested by centrifugation at 1000× *g* for 5 min. The pellets were lysed, and the RNA was isolated following the instructions of the RNeasy Mini Kit (Qiagen GmbH, Hilden, Germany). The RNAs were subjected to electrophoresis at 16 mA in a 7.0 M urea/5% (*w*/*v*) polyacrylamide gel for 120 min and stained with GelRed nucleic acid staining [38].

### 3.8. DNA Fragmentation Analysis

COLO 320 cells (1 × 10^6^/plate) were incubated for 72 h in the presence of eryngitins (300 nM). After treatment, cells were harvested by centrifugation (1000× *g* for 5 min). The pellets were lysed in 50 mM Tris•Cl, pH 8.0, containing 10 mM EDTA and 0.5% SDS, and the DNA was isolated following the manufacturer’s instructions (Genomic Prep Cells and Tissue DNA Isolation Kit (GE Healthcare, Madrid, Spain)). DNA electrophoresis was carried out as previously reported [38].

### 3.9. Antifungal Activity

*P. digitatum* growth inhibition assay in the presence or absence of different concentrations of eryngitin 3 and 4 was performed in 96-well microtiter plates, as previously reported [38].

### 3.10. Reduction and S-Pyridylethylation

*S*-pyridylethylation (cysteinyl residues alkylation with 4-vinylpyridine [20]) with and without reducing agent was performed as previously reported [22]. Native or alkylated desalted proteins were obtained by RP-HPLC using a BioBasic-4 column (150 × 4.6 mm, 5-μm particle size; Thermo Fisher Scientific, Rodano, Italy) at 25 °C. Solvents used are Milli-Q water containing 0.1% TFA (solvent A) and acetonitrile containing 0.1% TFA (solvent B). Protein elution was performed using a linear gradient of solvent A and solvent B, from 5% to 65% of solvent B over 60 min (flow rate of 1.0 mL/min), monitoring the absorbance at 214 nm.

### 3.11. Automatic N-Terminal Edman Degradation

N-terminal sequence of the proteins was obtained by using automated Edman degradation performed on a Shimadzu PPSQ 33B sequencer (Shimadzu Italia S.r.l., Milan, Italy) through the service of ‘Protein/Peptide sequencing’, Institute of Biosciences and Bioresources (IBBR-CNR, Naples, Italy). Proteins were firstly alkylated with 4-vinylpyridine, desalted by RP-HPLC (Section 3.10), freeze-dried and loaded on the sequencer.

### 3.12. Protein Digestion for MALDI-ToF Analysis

Enzymatic hydrolyses (i.e., trypsin TPCK-treated, chymotrypsin, endoproteinase Glu-C and pepsin) of eryngitin 3 and 4 (50 µg) were achieved by dissolving the alkylated proteins in appropriate buffer. Specific conditions for the different proteases are listed below:

Trypsin: buffer 50 mM (NH_4_)HCO_3_, containing 10% acetonitrile; enzyme/substrate 1:50 (*w*/*w*) final ratio added in three steps (the first at 0 h (E/S: 1:200), the second at 4 h (E/S: 1:100) and the third at 12 h (overnight, E/S: 1:50)) for 16h total; incubation at 37 °C [22,38];

Endoproteinase Glu-C: buffer Na-phosphate 50 mM, pH 7.8, containing 10% acetonitrile; enzyme/substrate 1:25 (*w*/*w*) final ratio added in three steps (the first at 0 h (E/S: 1:100), the second at 4 h (E/S: 1:50) and the third at 12 h (overnight, E/S: 1:25)) for 16 h total; incubation at 25 °C [22,38];

Chymotrypsin: buffer 50 mM (NH_4_)HCO_3_, containing 10% acetonitrile; enzyme/substrate 1:50 (*w*/*w*) final ratio added in two steps (the first at 0 h (E/S: 1:100) and the second at 2 h (E/S: 1:50)) for 4 h total; incubation at 37 °C [22,38];

Pepsin: buffer 0.2 M KCl/HCl, pH 1.3; enzyme/substrate 1:100 (*w*/*w*) final ratio added in two steps (the first at 0 h (E/S: 1:200) and the second at 2 h (E/S: 1:100)) for 4 h total; incubation at 37 °C [40,41].

### 3.13. MALDI-ToF Mass Spectrometry Analyses

Relative molecular mass (M*r*) of eryngitin 3 and 4 (native or *S*-pyridylethylated with and without reducing pre-treatment), as well as peptides from the enzymatic hydrolyses, were determined by using a MALDI-ToF mass spectrometer (Waters Micromass Co., Manchester, UK), as previously reported [38]. The samples (1 μL) were mixed with 1 μL of saturated α-cyano-4-hydroxycinnamic acid matrix solution (10 mg/mL) in acetonitrile: 0.1% TFA in water (1:1, *v*/*v*). Afterwards, a droplet of the resulting mixture (1 μL) was placed on the mass spectrometer’s sample target and dried at room temperature. Once the liquid was completely evaporated, samples were loaded into the mass spectrometer and analyzed in positive ion mode. In reflectron mode, the instrument was externally calibrated using a tryptic alcohol dehydrogenase digest (Waters, Milford, MA, USA). For linear mode, a 4-point external calibration was applied using an appropriate mixture (10 pmol/μL) of insulin, cytochrome C, horse myoglobin and trypsinogen as standard proteins (Sigma-Aldrich, Milan, Italy). A mass accuracy near the nominal (50 and 300 ppm in reflectron and linear modes, respectively) was achieved for each standard. All the spectra were processed and analyzed by using the software MassLynx 4.1 (Waters, Milford, MA, USA).

### 3.14. Sequence Analyses

Amino acid sequence identical to N-terminal sequence of eryngitin 3 and 4 was retrieved by using BlastP (https://blast.ncbi.nlm.nih.gov/Blast.cgi?PAGE=Proteins; accessed on 3 May 2023). To this aim, eryngitin 3 N-terminal amino acid sequences were used as queries by setting non-redundant protein sequences (nr) database and Fungi (taxid:4751) as organisms. Sequences alignment was performed using ClustalW tool available online (https://npsa-prabi.ibcp.fr/cgi-bin/npsa_automat.pl?page=npsa_clustalw.html; accessed on 3 May 2023). Similarity/identity matrix and Logo were obtained using the Sequence Identity and Similarity (SIAS) tool (http://imed.med.ucm.es/Tools/sias.html; accessed on 3 May 2023) and WebLogo-3 (https://weblogo.threeplusone.com/create.cgi; accessed on 3 May 2023), respectively. Information on hypothetical protein BDN71DRAFT_1455417 (GenBank AC: KAF9489889.1) annotation is reported in the following link (https://mycocosm.jgi.doe.gov/cgi-bin/dispGeneModel?db=Pleery1&id=1455417; accessed on 3 May 2023) and refers to the nucleotide sequence present in *P. eryngii* ATCC 90797 genome [24], named ‘jgi|Pleery1|1455417’.

Three-dimensional structural models of eryngitin 3 and 4 were obtained using AlphaFold Structure Prediction Database (https://www.alphafold.ebi.ac.uk/; accessed on 9 May 2023) [42] and available in UCSF ChimeraX software version 1.6.1 [43,44]. Studies on electrostatic superficial regions and graph representations were performed by using UCSF Chimera suite version 1.16 (https://www.cgl.ucsf.edu/chimera/; accessed on 9 May 2023) [45].

## 4. Conclusions

Knowledge of a protein’s primary structure may contribute to understanding biological differences in the enzymatic and functional activities of proteins.

In light of this, we determined the primary structures of eryngitin 3 and 4, two ribotoxin-like proteins isolated from *P. eryngii* fruiting bodies. Despite both enzymes being able to release the α-fragment (a hallmark of RL-Ps from edible mushrooms) when incubated with ribosomes from different sources (yeast and mealworm insect), they exhibit different biological and antipathogenic activities. Eryngitin 3 and 4 consist of 132 amino acid residues with a single free cysteinyl residue. Most of the residues between the two toxins are identical, considering that the two proteins have the same amino acid sequence, apart from a single residue at both the N-terminus (additional phenylalanyl residue for eryngitin 3), absent in eryngitin 4, and the C-terminus (additional arginyl residue for eryngitin 4), absent in eryngitin 3. In addition, the predicted models of the two toxins by AlphaFold highlight that the additional positive charge retrieved from eryngitin 4 at the C-terminal region could interfere with the binding to the substrate (SRL) or with some ribosomal proteins (e.g., P-stalk) during substrate recognition, providing a possible explanation for their different toxicity.

Finally, considering the presence of a single CDS in the *P. eryngii* genome, the two eryngitins could be the distinct products of the same transcript subjected to two different post-translational processes (e.g., proteolytic events) necessary to obtain the two enzymes.

Further studies will be necessary to understand the function of eryngitin 3 and 4 (both constitutively expressed in *P. eryngii* fruiting bodies), likely using a different action such as self-defence (i.e., the more toxic eryngitin 3) or for a physiological role (i.e., the less toxic eryngitin 4).

## Data Availability

The data presented in this study are available on request from the corresponding author.

## Supplementary Materials

The following supporting information can be downloaded at https://www.mdpi.com/article/10.3390/ijms241914435/s1.
