# Supplementary material for: The Biological Action and Structural Characterization of Eryngitin 3 and 4, Ribotoxin-like Proteins from Pleurotus eryngii Fruiting Bodies
Source: Int J Mol Sci. 2023 Sep 22;24(19):14435. doi: 10.3390/ijms241914435 (PMC10572553; doi:10.3390/ijms241914435)
Supplement: Supplementary file 1 [file ijms-24-14435-s001.zip › ijms-2603301-SI.pdf]

SUPPLEMENTARY MATERIALS

The Biological Action and Structural Characterization of Eryngitin 3 and 4, Ribotoxin-like Proteins from *Pleurotus eryngii* Fruiting Bodies

Sara Ragucci [1], Nicola Landi [1,2], Lucía Citores [3], Rosario Iglesias [3], Rosita Russo [1], Angela Clemente [1], Michele Saviano [2], Paolo Vincenzo Pedone [1], Angela Chambery [1], José Miguel Ferreras [3] and Antimo Di Maro [1,*]

[1] Department of Environmental, Biological and Pharmaceutical Sciences and Technologies (DiSTABiF),
University of Campania 'Luigi Vanvitelli', Via Vivaldi 43, 81100 Caserta, Italy;
sara.ragucci@unicampania.it (S.R.); nicola.landi@unicampania.it (N.L.);
rosita.russo@unicampania.it (R.R.); angela.clemente@unicampania.it (A.C.);
paolovincenzo.pedone@unicampania.it (P.V.P.);
angela.chambery@unicampania.it (A.C.)

[2] Institute of Crystallography, National Research Council, Via Vivaldi 43, 81100 Caserta, Italy;
michele.saviano@cnr.it

[3] Department of Biochemistry and Molecular Biology and Physiology, Faculty of Sciences,
University of Valladolid, E-47011 Valladolid, Spain; lucia.citores@uva.es (L.C.); riglesias@uva.es (R.I.);
josemiguel.ferreras@uva.es (J.M.F.)

* Correspondence: antimo.dimaro@unicampania.it; Tel.: +39-0823-274409

Table S1. Cytotoxicity of eryngitin 3 and 4. HeLa or COLO 320 cells were grown in RPMI 1640 medium and incubated with different toxin concentrations for 48 or 72 h, and cell viability was evaluated by a colorimetric assay, as indicated in Materials and Methods section. Data represent the mean of IC_{50} (the concentration of toxins causing a 50% reduction in viability) obtained from two experiments performed in duplicate.

		IC_{50} (nM)	
Cell line	Time (hour)	Eryngitin 3	Eryngitin 4
HeLa	48	15	640
HeLa	72	3.0	230
COLO 320	48	1200	>3100
COLO 320	72	420	1700

Table S2. Experimental relative molecular masses of tryptic (T), endoproteinase Glu-C (GC), pepsin (P) and chymotryptic (C) peptides from eryngitin 4 determined by MALDI-ToF mass spectrometry.

Peptide	Sequence position	Experimental molecular mass [a]	Theoretical molecular mass	Δ (Da)	Missed cleavage at	Notes
trypsin peptides						
T-1	1-10	1122.54	1122.54	-		*N-terminal*
T-2	16-22	855.43	855.42	0.01		
T-3	23-35	1533.94	1533.83	0.11	K28	
T-3’ [b]	29-35	851.50	851.50	-		Cys-PE
T-4	36-40	563.27	563.32	0.05		
T-5	41-50	1043.61	1043.60	0.01		
T-6	61-72	1237.61	1237.60	0.01	K66	
T-7	73-79	786.42	786.42	-		
T-8	97-103	699.39	699.41	0.02		
T-9	104-108	681.34	681.36	0.02	K105	
T-10	110-120	1269.73	1279.72	0.01	K115	
T-10’ [b]	116-120	586.24	586.29	0.05		
T-11	121-132	1465.82	1465.74	0.08		*C-terminal*
endoproteinase Glu-C peptides						
GC-1	1-11	1252.60	1251.58	1.02	E2	*N-terminal*
GC-1’ [b]	3-11	1065.56	1065.52	0.04		
GC-2	12-24	1374.82	1377.72	2.90		
GC-3	105-112	1049.61	1049.63	0.02		
GC-4	113-132	2361.86	2361.22	0.64		*C-terminal*
pepsin						
P-1	26-48	2502.45	2502.43	0.02	*alternative cleavage*	
P-1’ [b]	32-41	1127.61	1127.59	0.02	*alternative cleavage*	
P-1’’ [b]	32-45	1568.85	1568.82	0.03	*alternative cleavage*	
P-1’’’ [b]	32-47	1724.93	1724.91	0.02	*alternative cleavage*	
P-2	39-59	2235.29	2236.20	0.91	*alternative cleavage*	
P-3	52-61	1088.59	1088.58	0.01	*alternative cleavage*	
P-3’ [b]	59-77	2061.09	2061.04	0.05	*alternative cleavage*	
P-3’’ [b]	68-79	1244.69	1244.68	0.01	*alternative cleavage*	
P-4	79-88	1107.48	1107.57	0.09	*alternative cleavage*	
P-5	83-96	1459.83	1459.77	0.06	*alternative cleavage*	

P-6	112-124	1588.86	1588.85	0.01	*canonical cleavage*	
P-6’	114-124	1372.82	1372.78	0.04	*alternative cleavage*	
P-7	125-132	920.43	920.44	0.01	*canonical cleavage*	*C-terminal*
P-7’ [b]	124-132	1067.53	1067.50	0.03	*alternative cleavage*	*C-terminal*
P-7’’ [b]	112-132	2491.47	2490.27	1.2	F124; *canonical cleavage*	*C-terminal*
chymotrypsin						
C-1	1-12	1364.59	1364.67	0.08		
C-2	21-29	113.49	113.56	0.07		
C-3	32-42	1314.55	1313.66	0.89	Y32	
C-4	43-76	3535.75	3532.93	2.82	average	
C-5	99-107	1049.45	1049.54	0.09		
C-6	108-123	1924.99	1924.14	0.85	L111	
C-6’ [b]	112-123	1441.70	1441.78	0.08		
C-7	127-132	548.11	548.28	0.17		*C-terminal*

[a] $[M+H^+]^+$ experimental molecular mass values obtained by MALDI-ToF MS. The monoisotopic molecular masses have been considered, except for the C-4 peptide for which the average molecular mass is reported.
[b] Not reported in Fig. 4A.

Table S3. Experimental molecular mass values of tryptic (T), endoproteinase Glu-C (GC), pepsin (P) and chymotryptic (C) peptides from eryngitin 3 determined by MALDI-ToF mass spectrometry.

Peptide	Sequence position	Experimental molecular mass [a]	Theoretical molecular mass	Δ (Da)	Missed cleavage at	Notes
trypsin peptides						
T-1(a)	1-16	1798.25	1797.90	0.35	K11	*N-terminal of component a in eryngitin 3*
T-1' [b]	1-11	1269.75	1269.61	0.14		*N-terminal of component a in eryngitin 3*
T-1(b)	2-11	1122.59	1122.54	0.05		*N-terminal of component b in eryngitin 3*
T-2	17-23	855.45	855.42	0.03		Cys-PE
T-3	24-36	1533.96	1533.83	0.13	K29	
T-4	37-41	563.29	563.32	0.03		
T-5	62-73	1237.63	1237.59	0.04	K67	
T-6	74-80	786.44	786.43	0.01		
T-7	98-104	699.41	699.41	-		
T-8	105-110	808.42	809.45	1.03	K106 and K109	
T-9	110-121	1397.88	1397.81	0.07	K110 and K116	
T-9' [b]	110-116	830.56	830.53	0.03	K110	
T-10 [b]	117-121	586.26	586.29	0.03		
endoproteinase Glu-C peptides						
GC-1	1-12	1398.74	1398.65	0.09	E3	*N-terminal of component a in eryngitin 3*
GC-1' [b]	4-12	1065.54	1065.52	0.02		
GC-2	13-25	1375.82	1377.72	1.90		
GC-3	106-113	1049.67	1049.63	0.04		
GC-4	114-132	2206.68	2205.12	1.56		*C-terminal*
pepsin						
P-1	14-32	2044.09	2044.13	0.04	L30; *canonical cleavage*	Cys-PE
P-2	33-46	1568.85	1568.82	0.03	*alternative cleavage*	
P-2' [b]	33-48	1724.96	1724.91	0.05	*alternative cleavage*	
P-3	45-63	1973.15	1973.07	0.08	*alternative cleavage*	
P-4	83-96	1459.85	1459.77	0.08	*alternative cleavage*	

P-5	112-124	1588.89	1588.85	0.04	*canonical cleavage*	
P-5’ [b]	115-125	1372.82	1372.78	0.04	*alternative cleavage*	
P-5’’ [b]	120-125	817.44	817.47	0.03	*alternative cleavage*	
chymotrypsin						
C-1	31-43	1525.95	1525.82	0.13	L32 and Y33	
C-2	43-76	3537.49	3532.93	4.56	average	
C-3	100-108	1049.47	1049.54	0.07		
C-4	109-124	1925.42	1924.14	1.28	L112	
C-4’ [b]	113-124	1441.78	1441.78	--		

[a] $[M+H^+]^+$ experimental molecular mass values obtained by MALDI-ToF MS. The monoisotopic molecular masses have been considered, except for the C-2 peptide for which the average molecular mass is reported.

[b] Not reported in Fig. 4B.

Subscripts letters in brackets for peptide T-1 indicate the two components (a and b) retrieved in different amount in eryngitin 3 mixture after RP-HPLC, see main text.

Figure S1

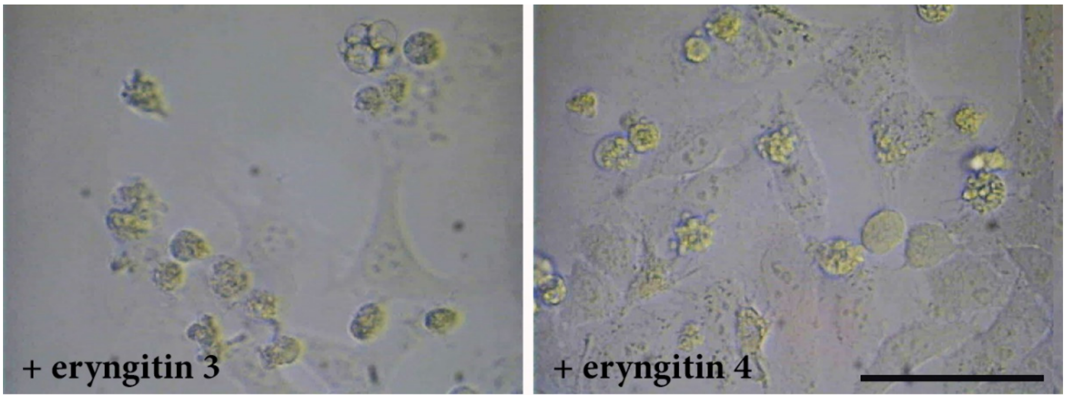

Figure S1. Morphological changes visualized microscopically when HeLa cells were incubated with eryngitin 3 (0.3 µM) or 4 (3.0 µM) after 48 h of incubation. Bar, 50 µm.

Figure S2

```
                 1          10          20          30          40          50
                 •          •           •           •           •           •
                 MSSDQAEVCA AEAVVFGEVT  QNYPSKELAS  KAACTWAKID  DPNKLVLYTS
                 eryngitin 4     GEVT   QNYPSKELAS  KAACTW
                             a [ FGEVT  QNYPSKELAS  KAACT-
               eryngitin 3   b [  GEVT  QNYPSKELAS  KAACTW

                51          60          70          80          90          100
                 •          •           •           •           •           •
                 RVGPYKGWVV GVGITRSSGT  IEDIVRVDSD  DKTGTATKGI  HFNAKNSKDS

               101          110         120         130         140         150
                 •          •           •           •           •           •
                 SQTLAAVIEP TKALTPAARD  KQYKKILESI  KGQEPRVIWF  WWSTGAGRFA

               151          160
                 •          •
                 ELDLEDATED AA
```

Figure S2. Amino acid sequence of hypothetical protein BDN71DRAFT_1455417 (AC: KAF9489889.1) retrieved in *P. eryngii* genome used as a reference protein for structural characterization of eryngitin 4 and 3 by MALDI-ToF MS analysis and peptides mapping. In addition, the experimental N-terminal amino acid sequences of both eryngitins obtained by automatic Edman degradation are reported. For eryngitin 3, two N-terminal amino acid sequences, named component a and b, were detected.

Figure S3

```
1                         start codon                                   60
(80703) agt tcg aat tcc cag atg tct tct gat caa gct gaa gtc tgc gcc gcc gag gcg gta gtc
                             M   S   S   D   Q   A   E   V   C   A   A   E   A   V   V
61                                                                     120
ttc ggt gag gtg acg cag aac tac cca agc aaa gag tta gct agt aaa gca gcc tgt acc
 F   G   E   V   T   Q   N   Y   P   S   K   E   L   A   S   K   A   A   C   T
      |--------------> start of eryngitin-4
 |--------------> start of eryngitin-3
121                                                                    180
tgg gct aag att gac gat ccg aac aag ctc gtg ttg tac acc ag gta ttg gtc taa tga c
 W   A   K   I   D   D   P   N   K   L   V   L   Y   T   S
181                                                                    240
                 intron 1
c cct tgt cac tgt cag cgc gct gac cta ctc ttt ag t cga gtt ggt ccg tac aaa gga tg
                                                   R   V   G   P   Y   K   G   W
241                                                                    300
                              intron 2
g gtt gtc g gta cgg gct tct gtg ata act gcc ttc acg atg act aac tat gct gaa g gt g
   V   V                                                                 G
301                                                                    360
tt ggc atc acg cgt tct agc ggc acc ata gaa gac att gtt cgc gtc gat tcc gat gac a
V   G   I   T   R   S   S   G   T   I   E   D   I   V   R   V   D   S   D   D
361                                                                    420
aa acg gga acg gcc acc aaa ggc atc cac ttc aac gcc aag aac tct aaa gac tct tcg c
K   T   G   T   A   T   K   G   I   H   F   N   A   K   N   S   K   D   S   S
421                                                                    480
ag acc ctt gcc gcc gtc atc gag cca acg aag gcg ctc acc ccc gcc gcc cgg gac aaa c
Q   T   L   A   A   V   I   E   P   T   K   A   L   T   P   A   A   R   D   K
481                                                                    540
ag tac aag aag atc ctg gaa tct atc aag ggc cag gaa ccg agg gtg att tgg ttt tgg t
Q   Y   K   K   I   L   E   S   I   K   G   Q   E   P   R   V   I   W   F   W
541                                                                    600
gg agt acg gga gcg gga agg ttc gca gaa ctt gat ctt gaa gat gcg acg gag gat gct g
W   S   T   G   A   G   R   F   A   E   L   D   L   E   D   A   T   E   D   A
        <--------------| end of eryngitin-4
601  <--------------| end of eryngitin-3                               660
ct tga gta tga cgt taa gta att atc atg cct cag acg tgc gac att tcc ttg cct cgg t
A   -
661                                                                    720
gg tgg tga gcg ttc tcg ctt cgt cag agg tac ctc gtt gat atc gct tca tac cca tcc c
721                                                                    780
at ctg ctg gca cat agc tct gtg tgt ggt cag gga ggg aac gac tat gag taa aaa acc a
781                                                        831
tt ata gac caa ccc tgc gtt ttc ctt cgt aat gat ggg ccg cat ttc gct g (81533)
```

Figure S3. Nucleotide sequence (831 bp) of ‘jgi|Pleery1|1455417’, present in *P. eryngii* ATCC 90797 genome, containing transcript region (730 bp) of both eryngitins. The ‘atg’ codon is highlighted by the green arrow; exons, introns and splice junctions are reported in black, green or underlined, respectively. The first (start) and last (end) residues of both purified eryngitin 4 and 3 are shown. Numbers in round brackets are referred to the specific region of scaffold_136:80703-81533 in *P. eryngii* genome.

Figure S4

```
                1                   20                  40                  60
                •                   •                   •                   •
eryngitin-4     -GEVTQNYPSKELASKAACTWAKI----DDPNKLVLYTSRVGP--YKGWVVGVGITRSSG
eryngitin-3     FGEVTQNYPSKELASKAACTWAKI----DDPNKLVLYTSRVGP--YKGWVVGVGITRSSG
ostreatin       -GEVTQNYPSKELASKAACTWAKV----DDPNKLVLYTSRVGP--YKGWVVGVGITRPSG
ageritin        -AETVQYYNSYSDASIASCAFVDSGKDKIDKTKLVTYTSRLAASPAYQKVVGVGLKTAAG
                 .*..* * * . ** *:*::..      * .*** ****:.       *****:.  :*
```

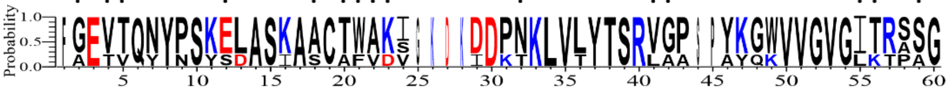

```
               61                   80                  100                 120
                •                   •                   •                   •
eryngitin-4     TIEDIVRVDSDDKTGTATKGIHFNAKNSKDSSQTLAAVIEPTKALTPAARDKQYKKILES
eryngitin-3     TIEDIVRVDSDDKTGTATKGIHFNAKNSKDSSQTLAAVIEPTKALTPAARDKQYKKILES
ostreatin       TIEDIVRVDSDDKTGTATKGIHFNAKNSKDSSQTLAAVIEPTKALTPAARDKQYKKILES
ageritin        SIVPYVRLDMDN----TGKGIHFNATKLSDSSAKLAAVLKTTVSMTEAQRTQLYMEYIKG
                :*   **:* *:    : *******.: .*** .****:: * ::* * * : * : ::.
```

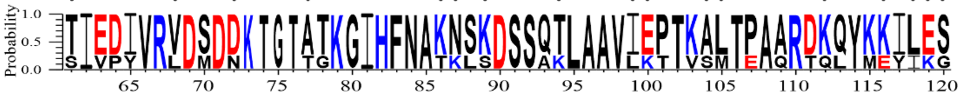

```
               121                  140
                •                   •
eryngitin-4     IKGQEPRVIWFWWSTGAGR-
eryngitin-3     IKGQEPRVIWFWWSTGAG--
ostreatin       IKGQEPRVIWLWWSTGAG--
ageritin        IENRSAQFIWDWWRTGKAPA
                *:.:. :.** ** ** .
```

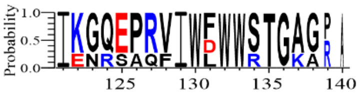

Figure S4. Amino acid sequence alignment of eryngitin 4, eryngitin 3, ostreatin and ageritin. Ostreatin and ageritin are two well-characterized RL-Ps isolated from *P. ostreatus* and *C. aegerita* fruiting bodies, respectively. The standard one-letter code was used for the amino acid residues. Identical residues (*), conserved substitutions (:) and semiconserved substitutions (.) are reported. Amino acid residues of the catalytic site and the single free cysteinyl residue are reported in cyan and green, respectively. Among the amino acid blocks, the Logo representation of alignment is reported. Letter height is proportional to the conservation of that amino acid at that position in the alignment with respect to all the amino acids; letter width is proportional to the conservation of that amino acid but includes gaps. In red and blue, the residues with negative or positive charges, respectively.

Figure S5

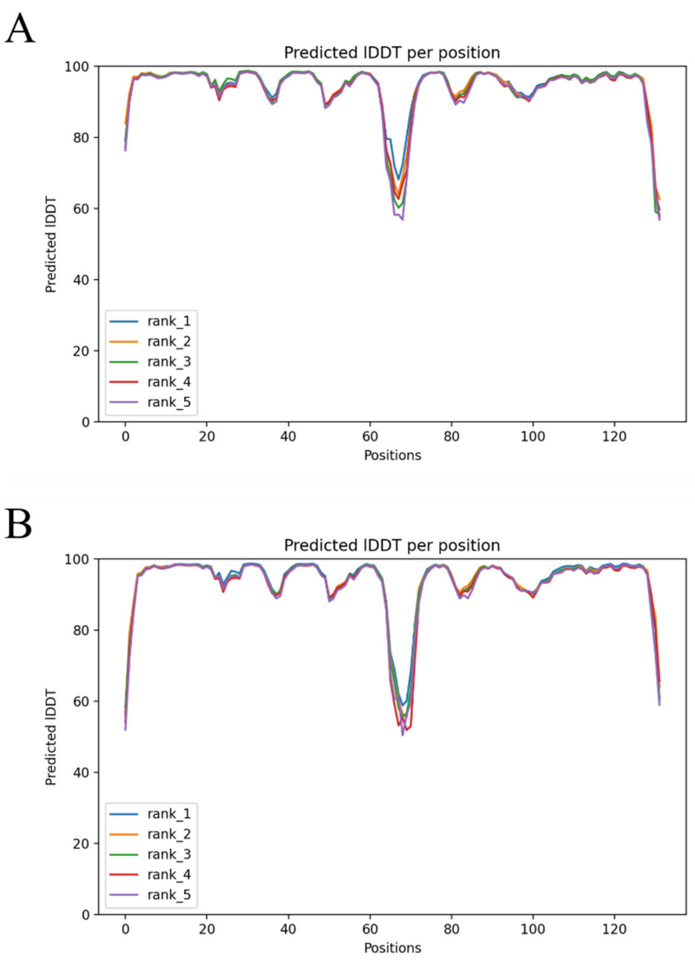

Figure S5. In (**A**) and (**B**), graphical representations of the Local Distance Difference Test (lDDT) of eryngitin 4 and 3, respectively. IDDT is a superposition-free score robust tool used to evaluate the local distance differences of all atoms in the resulting model, including validation of stereochemical plausibility. In particular, the graph should be interpreted as follows: i) regions with pLDDT > 90 are expected to be modelled to high accuracy; ii) regions with pLDDT between 70 and 90 have expected to be modelled well; iii) regions with pLDDT between 50 and 70 are low confidence and should be treated with caution; and iv) regions with pLDDT < 50 have often a ribbon-like appearance and should not be interpreted.
